# Supplementary material for: Identification and selection of optimal reference genes for qPCR-based gene expression analysis in Fucus distichus under various abiotic stresses
Source: PLoS One. 2021 Apr 28;16(4):e0233249. doi: 10.1371/journal.pone.0233249 (PMC8081170; doi:10.1371/journal.pone.0233249)
Supplement: S1 Fig — Each reference gene was matched to a Fucus transcriptome gene (Trinity gene CDS; Linardić et al., 2020) and PCR amplicons resulting from the PCR amplification with the designed primers in Table 1. (PDF) [file pone.0233249.s001.pdf]

EF1alpha

Amplicon:

ATGAGGTGGGCCATCTACCTGAAGAAGGTGGGCTACAAACCTATCAAGATTCCCTTCGTGCCCATCTCGGGTTGGGC
CGGCGACAACATGATCGACAGGTCCCCCAACATGCCTTGGTA

Fucus transcriptome ‘gene’ CDS (TRINITY_DN35856_c3_g5_i2):

GAGTTCGAGGCCGGTATCTCGAAGAACGGCCAGACCCGCGAGCACGCGCTTCTGGCTTACACGCTTGGCGTGAAGCA
GATGATCGTGTGCGTTAACAAGATGGACGACTCTTCCGTGATGTACGGCGAGGCCCGCTACAAGGAGATCAAGGATG
AGGTGGCCATCTACCTGAAGAAGGTGGGCTACAAACCCATCAAGATTCCCTTCGTGCCCATCTCGGGCTGGGCCGGC
GACAACATGATCGACAGGTCCCCCAACATGCCTTGGTACAAGGGCCCTTTCCTGCTTGAGGCCCTCGACAACTGTAA
GGAGCCTACGCGCCCCACGGACAAGCCCCTTCGTCTCCCACTGCAAGACGTGTACAAGATCGGTGGTATCGGCACGG
TTCCCGTCGGCCGCGTGGAGACCGGCGTCCTTAAGCCCGGTATGGTGGTGACCTTCGCGCCGGTGAACCTTGAAACC
GAAGTGAAGTCTGTGGAGATGCACCACGAGTCTTTGCCGGAGGCTTTGCCCGGAGACAACGTGGGCTTCAACGTGAA
GAACGTGTCGGTGAAAGACATCCGCCGAGGGTACGTGGCTGGTGCCACTAAGAAGGACCCTCCTCTGGGTGCCTCCA
TGTTCAGCGCACAGGTTATCGTCATGAACCACCCCGGCCAGATCTCCAACGGCTACGCACCAGTGCTGGACTGCCAC
ACTGCGCACGTGGCGTGCAAGTTCAAGGAGATCACCCAGAAGATGGACCGTCGATCTGGTAAGATCATGGAGGAAGG
CCCCAAGTTCGTGAAGACGGGCGACGCGTGCATGGTGAACATGGAGCCGTCCAAGCCCATGTGCGTGGAGTCCTTCG
CCGAGTACCCGCCTCTGGGACGCTTCGCCGTGCGCGACATGCGTCAGACTGTGGCCGTGGGCGTGATCAAGTCCGTC
GAGAAGAAGGCGCCCACCAAGAAGGGCAAGAAGTAG

UBCE2

Amplicon:

AAGCTCAACATGGGCTGTGTCGACCAGAGAAGCGGCGAGGTACAACGACACAGGCTTCAGGTGCTGGCCCAGTGGAA
CAGGAATTACGGCATTGAGCAGGTACTGGTGGC

Fucus transcriptome ‘gene’ CDS (TRINITY_DN33985_c0_g2_i1):

ATGACAACTGCGACCGGAGAGGTTATTATGGTCCCGCGCAACTTCAAGCTGTTGGAGGAGCTCGAGAAGGGCGAAAA
GGGCGTGGGGGATCACAGCGTGAGCTTTGGCCTGGTGGACGGGGAGGATATCTTCATGACCGACTGGAACGGAACCG
TCCTTGGACCGAACGGGACTCCGCACGAGGGCCGGCTCTACACGTTGAGGATTCACTGCGGAGAGAACTATCCCGAT
CAGTGCCCGCACGTGCGCTTCGTGAGCAAGCTCAACATGGGCTGTGTCGACCAGAGAAGCGGCGAGGTACAACGACA
CAGGCTTCAGGTGCTGGCCCAGTGGAACAGGAATTACGGCATTGAGCAGGTACTGGTGGCCCTAAGGAACGAGATGG
CTGGTCCCTCGAACCGCCGACTCCCCCAGCCGCCCGAGGGCACTGAGTTCTAG

ACT

Amplicon:

GACCTTTACGGCAACATCGTACTCTCCGGCGGTACCACCATGTTCCCCGGCATCGGCGAGCGTATGACCAAGGAGCT
TACGGCACTGGCACCTTCGACAATGAAGATCAAGGTTGTGGCACCA

Fucus transcriptome ‘gene’ CDS (TRINITY_DN37559_c3_g2_i3):

AAGGCCAACAAGGAGCGCATGACTCAGATCATGTTCGAGACCTTCAACGTGCCCGCTATGTACGTCAACATCCAGGC
CGTTCTCTCCCTCTACGCCTCCGGTCGTACCACCGGGTGTGTGCTCGATTCGGGTGACGGAGTGTCCCACACCGTGC
CCATCTACGAGGGGTACGCTCTACCCCACGCAATCAACCGCCTCGACCTCGCCGGGCGTGACCTGACCGATAACCTC
ATGAAGGTTTTGACCGAGCGTGGTTACTCCTTCACGACCACCGCGGAGCGCGAGATCGTTCGCGACATCAAGGAGAA
GCTCACCTACGTGGCGCTGGACTTCGACCAGGAGATGAAGACGGCCGCGGAGTCGTCTCAGCTTGAAAAGTCGTACG
AGCTCCCCGACGGAAACGTCATCGTGATCGGCAACGAGCGCTTCCGTTGCCCTGAGGTTCTCTTCCAGCCGTCGTTC
ATCGGAATGGAATCTTCGGGCATCCACGATTGCACCTTCAAGACCATCATGAAGTGCGACGTCGACATCCGTAAGGA
CCTTTACGGCAACATCGTACTCTCGGGCGGTACCACCATGTTCCCCGGCATCGGCGAGCGGATGACCAAGGAGCTGA
CGGCACTGGCACCTTCGACAATGAAGATCAAGGTTGTGGCACCACCCGAGCGGAAATACTCGGTGTGGATCGGTGGT
TCTATCCTCGCGTCGCTGTCCACTTTCCAGCAAATGTGGATTTCGAAGGCAGAGTACGATGAGTCTGGCCCGTCCAT
CGTTCACCGCAAGTGCTTCTAA

14-3-3

Amplicon:

CAAGATCGAGACAGAGTTGACGGACATCTGTGACGACATCCTGAAGATCATCGAAGCCGAGCTTATCCCGAACTCAA
CTTCCGAAGAGGGAAAAGTGTTTTACTACAAGATGAAGGGCGA CTACCACCGGTATCTTGCG

Fucus transcriptome ‘gene’ CDS (TRINITY_DN36694_c1_g1_i1):

ATGACCTCTCGCGATGATCTCGTTTACATGGCCAAGCTCGCTGAGCAGGCCGAGCGCTTTGATGAAATGGTGGACCA
CATGAAGGCTGTTGCTCAGCAGCCGAAGGAGCTTTCGGTTGAGGAGCGTAACCTCCTCTCCGTAGCGTACAAGAACG
TCATCGGCTCCCGTCGCGCCTCCTGGAGGGTGATCAGCTCCATTGAGGGCAAGGACACCGTGAGCGATCAGTTACCT

CTGATTCGTGACTACAAGTCCAAGATCGAGACAGAGTTGACGGACATCTGCGACGACATCCTGAAGATCATCGAGAA
CGAGCTTATCCCGAACTCAACCTCCGAAGAGGGGAAAGTGTTTTACTACAAGATGAAGGGCGACTACCACCGGTATC
TTGCGGAGTTCCAGTCCGCCGACGAACGGAAAACT
AGCGCCTCCGATGCCCTCGACGCATACCAGTCAGCCTCTGAGCTTGCAAACCAGGACCTTCCCCCGACCCACCCAAT
CCGCCTGGGTCTCGCCCTCAACTTCTCTGTCTTCTATTACGAGATCCTCAACTCCCCTGAGCGCGCATGCAACTTGG
CGAAGGCGGCATTCGACGACGCAATCGCAGAGCTGGACACGCTTTCTGAGGAATCTTACAAAGACTCGACACTTATC
ATGCAGCTCCTCCGGGACAACCTCACTCTGTGGACATCAGACCAGGGAGAGGCGGAGGAAGCACCTGGTAACGCCGA
CGGCACGGTGGTGGAGGACCTCTAA

40S

Amplicon:
ACGGCTGTCTGAACTTCACCGACGAAAACGACGAAGTGCTTATCTCCGGTTTCGGTCGCCGCGGTCACGCCGTGGGT
GATATCCCTGGTGTCCGTTTCAAGGTGGTGAAGGT

Fucus transcriptome ‘gene’ CDS (TRINITY_DN31154_c0_g1_i1):
ATGGGTAAGGGAAAGCCAGCAGGTATCCGCGCCGGGCGCAAGCTGCGGATTCACCGCCGCAACCAACGGTGGGCAAG
CAAGGACTACAACAAGTCTCACTCGGTTACGGCGATGAAGGCGAACCCTCTCGGTGGATCTTCCATGGCCAAGGGCA
TCGTCTTGGAGAAAATCGGTATCGAGGCCAAACAGCCCAACTCGGCCATCCGTAAGTGCGTAAGAGTACAGCTGATC
AAGAATGGGAAGAAGCTGGCGGCTTTCGTTCCGCGTGACGGCTGTCTGAACTTCACCGACGAAAACGATGAAGTGCT
GATCTCCGGTTTCGGTCGCCGCGGTCACGCCGTGGGTGATATCCCCGGTGTCCGTTTCAAGGTGGTGAAGGTCGCGG
GTGTGTCGCTCCTTGCGCTCTACAAGGACAAGAAGGAAAAGCCCCGCTCCTAA

EF1beta

Amplicon:
TTCGGAGTGAAGAAGCTCGTCTTGTCCTGTGTGGTCGAAGACGCCAAGGTTGGCGTCGATGACATCACCGACTGCAT
CGAGAAATTCGAAGACGAAGTCCAGTCGGTAGATATGACTACGATGAACCGCCTCTGA

Fucus transcriptome ‘gene’ CDS (TRINITY_DN38203_c1_g1_i5):
ATGAATGTGGATCGTGCTCAGTGGGAAAGTTCAGACACGGTGGACGTTGGGAGGGAAAATTGGTATCCGAACACAAT
CCGCTGCGTAACGGAGATCAAATGTCTTCACAGTACCCTGAGCCGCGCTGTCAGATACGCCAAGCTTTACCTGAAAG
ATTCCCCAAGTAACACAACAATGGGGCCTAAGTTCGACCTCGACACGCCCAAGGGCATCGGTGCGTTCAATGGCTTC
ATCTCATCCCGATCGTACGTAGAAGGCTACACCTTCTCCCAGGCCGACGCGGATATGTTCGCTACCTGTACGGCCAC
CCCGGACAAGTCGAAGGCGCCCCACGCCTACCGCTGGTTCATCCACATCGCCGCCCTTAAGGGGGTAATCAGCCCTT
CCTTGGCGCCGAGCAAAGAGTCGACGCCGGCGCCA
GAGCCTGCAACCGCACCACCAGCACCCGATTCCACCCCGAAGGAAGCACCTGCGGACGACGACGATATGGATGATAT
GTTCGACGACGATGAGGAGGAGGAGGCACCCAAGCCGGCGGAGAAGAGTCGCGCGGATAAAATGGCCGAGGCGAAGG
CGGCCAAGGATTCCAAGAAAAAGATCGACAAGTCCCAGATCGTGTTCGAGGTAAAGCCGTGGGAGGCGGGCGCCGAC
CTCAAGAGTCTTTTCGAGAAGATCAAGGAGGAGAAGATTGACGGCCTCGCGTGGGGTGAGGCCTACAAGCTGGTGCC
CGTGGCATTCGGAGTGAAGAAGCTCGTCTTGTCCTGCGTGGTCGAAGACGCCAAGGTTGGCGTCGACGACATCACCG
ACTGCATCGAAAAATTCGAAGACGAAGTCCAGTCT
GTAGACATGACTACGATGAACCGCCTCTGA

EF2alpha

Amplicon:
TGGACCACGGAAAGTCTACCCTGACGGATTCTCTTGTCTCGAAAGCCGGAATCATCGCCGCGAAGAATGCTGGCGAG
GCCCGCTTTACCGACACACGCCAGGACGAGCAGGACCGATGTATCACCATC

Fucus transcriptome ‘gene’ CDS (TRINITY_DN37573_c1_g1_i1):
CGCCCTTATTCATTCTGTGGCTGTACTAGGAGCACCCCTCATCAACCACACCCACGGACTTGGGCTCGCTTGCCTAC
CCCACCTTGTCGGAGCAGTCAGGCCGAGGCCCACAACTACCTTACCATGGTGAACTTCACGACAGACCAGCTGCGCG
AGATCATGGACAAGAAGAATAACATCCGTAACATGTCCGTGATTGCGCACGTGGACCACGGAAAGTCTACCCTGACG
GATTCTCTTGTCTCGAAAGCCGGAATCATCGCCGCCAAAAACGCTGGCGAGGCCCGCTTCACCGACACCCGCCAGGA
CGAGCAGGACCGATGTATCACCATCAAGTCCACCGGAATCTCAATGTTCTTCGAGTACAATCTGGATGCCGGAGAGA
AGGTTATGCGTGCCGAATTGGAGGCGAAGGCCTCGAAGAGTGCTGGGGAGACCGAGGAAGAGGCGAAGATCAGCGCT
GAGAAAGGTGTGGCCAAGTCTAAGGGTGATGATTCGGGCAAAAACGACAGAGTCAAGATCGACGATACTTCTTTCCT
CATCAACCTCATCGACTCTCCCGGCCACGTCGATTTCTCGTCCGAGGTGACCGCCGCACTTCGCGTTACCGATGGCG
CCCTCGTGGTTGTCGATTGCGTGGAAGGCGTTTGCGTGCAGACGGAAACCGTGCTCCGTCAGGCCATCTCGGAGCGC
GTAAGGCCTGTTCTCATGGTCAACAAGGTCGACCGTGCGCTGCTGGAGCTCCAACTACCCCCGGAGGAAATGTACCA
GTCTTTTGCCCGTGCCATCGAGTCGGTGAACGTGATCATCGCCACCTACAACGACGATTTGCTGGGTGACGTTCAGG

TCTACCCCGACATGGGTACTGTTGCCTTCGGGTCCGGGCTACACCAGTGGGGCTTCACCCTCAAGAAGTTCGCCAAG
ATCTATGCTGCCAAGTTCGGCATCCAGGAAGAGAAGATGATGCAAAAGCTGTGGGGCGACTGGTACTTTGACGCCGC
GGGAAAGAAGTGGAAGAAGTCGAGCGACAACGGCAAGCTGGAGCGCGCCTTCTGCCAGTGGATCATGTCTCCCATCT
GCAAGATGTTCGAGGCGATCATGGAGGATAAGAAGATTAAAATCCAAAAAATGCTCACGGCCGTGGGCGTCACCCTG
AAAGGAGAAGAGAAGCAACTCGTGGGCAAGCCGCTCCTCAAGCGCGTGATGCAGAAGTGGCTCCCCGCCGCGGACGC
GGTTCTGGAGATGATCGTGGTTCACCTACCCTCGCCGCCCCAGGCGCAGAAATACCGTGTTGAAAACTTGTACGATG
GTCCCCTTGATGACGAGGTGGCGCAATCGATCCGTACGTGCGACACCAGCCCCGGAGCACCCCTCTGCATGTATGTA
TCGAAGATGGTGCCGACCTCGGACAAGGGTCGCTTCTACGCTTTCGGCCGCGTGTTCGCTGGCACGATCGCTACCGG
ACAGAGGGTGCGTATCCTTGGGCCCAACTACGTGCCGGGCAAGAAGACCGACCTGTGGATCAAGAACATCCAGCGTA
CGATCATCATGATGGGCCGCTACGTAGAGCAGGTGCAGGACATCCCCGCGGGTAACACTTGCGGCCTCGTGGGTGTC
GACCAGTACCTCCTCAAGTCGGGTACGATCACTACCTCCGAGTCGGGCCACACAATCAAGACCATGAAGTTCAGCGT
GAGCCCCGTCGTTCGTGTGGCTGTGGAGCCGAAGAACCAGGCCGACCTGCCCAAGCTCGTCGAGGGAATGAAGCGGT
TATCGAAGTCCGACCCTATGGTTCTGTGCTACACGGAAGAGTCGGGCGAGCACATCATTGCTGGTTGCGGCGAGCTT
CACCTGGAGATCTGCCTTAAAGACCTCCAGGAGGACTTCATGGGGACGGAGGTCAAGATCTCTGACCCCGTTGTGTC
TTACCGCGAGACCGTCTCTGCTGAGTCGAGCCAAACGTGTCTGTCCAAGTCCCCCAACAAGCACAACCGTCTCTATG
TAACCGCTGGCCCACTGGAGTCTGGCGTTGCCGAGGACGTGGAAGAGGGTCGCCTTAACCCCCGTGATGATGCCAAG
ATCCGCGCGCGCTACCTGGCCGACACCCACGGTTGGGACGTGGGAGAGGCGCGTAAGATCTGGGCATTCGGCCCCGA
GGGCACGGGAACCAACATCTTTGTGGACGTTACCAAGGGTGTCAACTACCTGAACGAGATCAGGGAATCCGTGATCG
GCGGGTTCAACTGGGCCATGAACGAGGGACCCATGACGGAAGAGAAGGTGCGCGGGGTCAGGTTTAACCTCCTGGAT
GTGGTTCTACACGCTGACGCCATCCACCGTGGCATGGGCCAGATCATGCCCACCTCTCGCCGCGTGGTGTACGCGTC
CATGCTGACCGCGTCACCCGTGCTGCTCGAGCCGGTGTACCTATGCGAGATTTCCTGTCCTCAAGACGCGATGGGTG
GCTGCTACGGCGTTCTTACGAGGCGTCGCGGCCACGTGTTCGCCGAGGAGCAGCGCCCGGGAACCCCAATGATGCAA
TTGAGGGCTTACCTGCCCGTCATGGAATCTTTCGGCTTCACCGCTGACCTCCGGAAAAACACGGGCGGGAAGGCATT
CCCGCAAAATGTGTTCGACCACTGGCAGGAGATGAGCGGAAACCCGCAAGAAGCGGGTTCTAAAGCCTCAGACGTGG
TCTTGGGTGTGCGCAAGAGGAAGGGTCTCTCCGACTCAATCCCACCCCTCGACCGTTACCTCGACCGCTTGTAA

ARP2/3

Amplicon:

GGAAGCCTCTGGCTATTGGTCGTTTCTCCGGCAACTGGATGAGAAATCCCGCGGGCCGGCAA
AGCAAGCGGGANCCNTGNNCNCCATGGCTTCCGCCAGGGCGATGTTCCAAGCCAAGACNCAC

Fucus transcriptome ‘gene’ CDS (TRINITY_DN37000_c0_g2_i1):

ATGCCCCCGCAGAAGTACCAGGTTGTTTCGCCCTCGACAGGAATATCTTGCCACGCATGGAACGCCGACAAGTCCAT
GCTCGCGCTCTGCGCCAACGATAACCGTCTGCAGATCTACGAGGGATGCAACAGCCCGACCTTCTCGTCTTGGCGCC
TGGCGCACGTGCTGGAGGAGCACGACCTTTTGATCACGGGCATCTCGTGGTCACCTGTCACGAACAAGATAGTCACG
TGCTCGCACGACCGAAACGCCTTCGTGTGGTCATACGACGCTGCCCAGGGCAAGTGGAACCCAATTCTCTGCGCGCT
CAAGCTTAACCGGGCTGCCCTGGACGTGCAGTGGAGCCCAGCAGGAACAAAATTCGCCGTTGCGAGCGGCGCCAAGA
CGGTTGCCGTAAGCCGGCTCGACACCTCGCAGAGTACCGAGTGGTACGTGAGCAGGCTCATGAAGGGCCACAAGTCT
ACGGTCCAACAAGTGGCATGGCATCCCAACAATCTCGTCCTCGCCACGGCGTGCACGGACTTCAAGTGTCGTGTGGT
TTCGGCTGTGGTGGCAGAGGTCGATACCAATCCCAACCCAGCACCTTTCGGAACGGTCAAACCATTCGGCGAGGCGT
ACCACGTGTTCCCCTGCGCGGGTTGGGTGACGGCGGTAGCTTGGTCTCCCTCAGGGTCAACCCTGGCCTATGCAGGC
CAGGATTCGACGATTCACTTCATGCGCTTCGACCAGGGGAGCGCGGTGGTAGAGCAGAGGGTGCGGTACCCGCTCCT
GCCCGTGGGGTGCTTGACCTTCTTGAGCGAGAGGGCGGTTGTTGGTGGAGGGCACGACATGAACCCTTTGGTGTTCG
CGTCGGAAGCCTCTGGCTATTGGTCGTTTCTCCGGCGACTGGATGAGAAATCCCGCGGGCCGGCAAAGCAAGCGGGA
GCCGTGAGCTCCATGGCTTCCGCGAGGGCGATGTTCCAAGCCAAGACCACCCGGGGTCAGGCAGCCTCCGGCGGAGC
GGGTCAGGACCTGTGGACGCAGCATGCCAACTCCATCGTGGGGATGGAGAGCATGGGACACGCGGAAGACCCAACGT
GCTCGAAGTTTTCCACCTGCGGTTTGGACGGAAGGATAGTGGTATGGAATGTTCCCACTCTCGACATCGACATGCAA
GCCTTGGGGCTATGA

GAPDH

Amplicon:

TCTTGGGTTACACCGAGGACTCTGTGGTTTCCTCGGACTTCATTGGTGACCCTCGCTCGTCCATCCTGGACGCCAAC
GCCGGCATCGCCCTCAGTGACACCTTCGTCAAGCTCGTGTCGTGGTAC

Fucus transcriptome ‘gene’ CDS (TRINITY_DN36486_c2_g3_i1):

GACTATAATAGTAGAAGCAACATCGTGAAGAGCAAGGCGCAGAGATCTACACGCCGAAACAAGATGGTCAACGTTGC
TATCAACGGCTTTGGACGCATCGGGAGATTGGTTCTTCGTGCCGCCAAGCACAACAAGCACATCAATGTCGTGGCCG
TCAACGACCCCTTTGTACCCCCGGAGTACATGAATTACATGTTCAAGTACGACACCGTCCATGGCGTCTACGACGGC

ACCGTAGAGAACGACGACTCGAGCATCATCGTCGATGGGAAGAAGATCAAGGTGTTCGGCGAAATGGACGCCGCAAA
CGTCAAGTTCGGGGACGTAGGTGCCGACTACGTGGTGGAGAGCACTGGTCTCTTTACCACGACAGAATCAGCGGCCG
TGCATCTCAAGGGGGGAGCAAAAAAGGTGGTGATCAGCGCTCCCTCCCAAGACGCGCCTATGTTCGTGATGGGCGTC
AACCAGGACAAGTATACCCCCGACATGCACGTGGTCTCCAATGCCTCGTGCACCACCAACTGCCTCGCCCCCATCGC
CAAGGTTATTAACGACTCGTTCGGCCTGAAGGAGGGGCTCATGACCACCGTGCACGCTGTCACCGCAACGCAAAAGA
CCGTAGACGGCCCATCCAAGAAAGACTGGAGAGGCGGGAGGGCGGCCGGATTTAACATCATCCCGTCCTCCACGGGA
GCCGCCAAGGCGGTGGGCAAGGTTATCCCCGAGCTGAACGGTAAGCTGACGGGCATGTCGTTCCGGGTCCCAACCGC
CAACGTGTCCGTGGTGGACCTCACCTGCCGGCTAGAGAAGCCTGCGAGCTACGAGGATATCAAGGCCGCGATGAAGG
CTGCCTCGGAGGGACCCATGCATGGCATCTTGGGTTACACCGAGGACTCTGTGGTTTCCTCGGACTTCATTGGTGAC
TCTCGCTCGTCCATCCTGGACGCCAACGCCGGCATCGCCCTCAGCGACACCTTCGTCAAGCTCGTGTCGTGGTACGA
CAACGAATGGGGCTACTCCTGTCGCGTGCTGGACCTCATCACCCATATGGACAAGTCGTCTTGA
