# Supplementary figures and images for: Identification and selection of optimal reference genes for qPCR-based gene expression analysis in Fucus distichus under various abiotic stresses
Source: PLoS One. 2021 Apr 28;16(4):e0233249. doi: 10.1371/journal.pone.0233249 (PMC8081170; doi:10.1371/journal.pone.0233249)

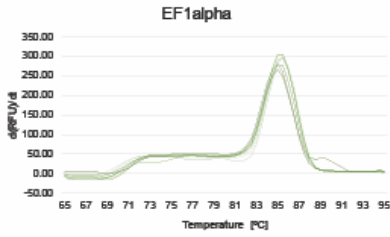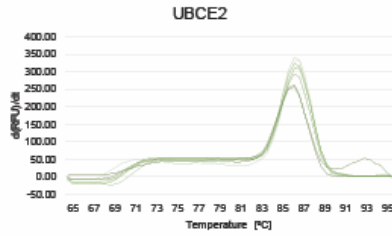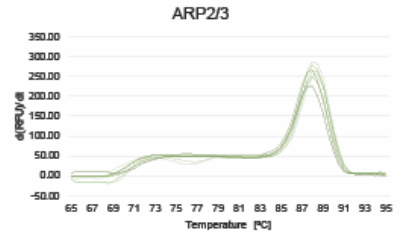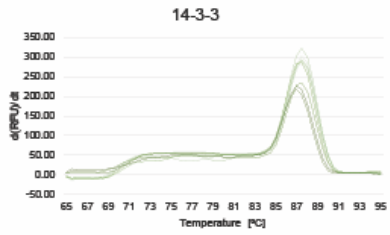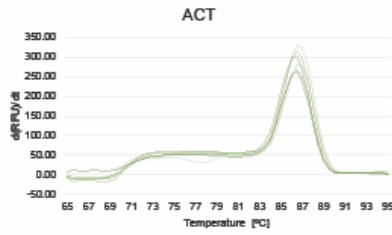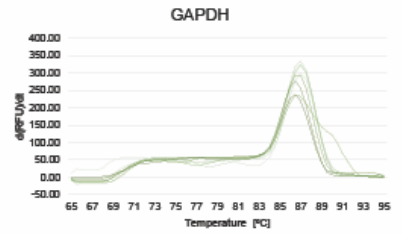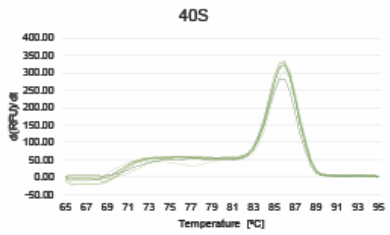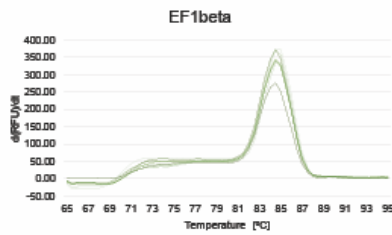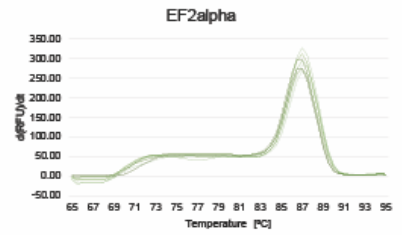

Supplement: S2 Fig — -ΔF/ΔT (change in fluorescence/change in temperature) is plotted against temperature to obtain a clear view of the melting dynamics of each reference gene. (PDF) [file pone.0233249.s002.pdf]

Cq

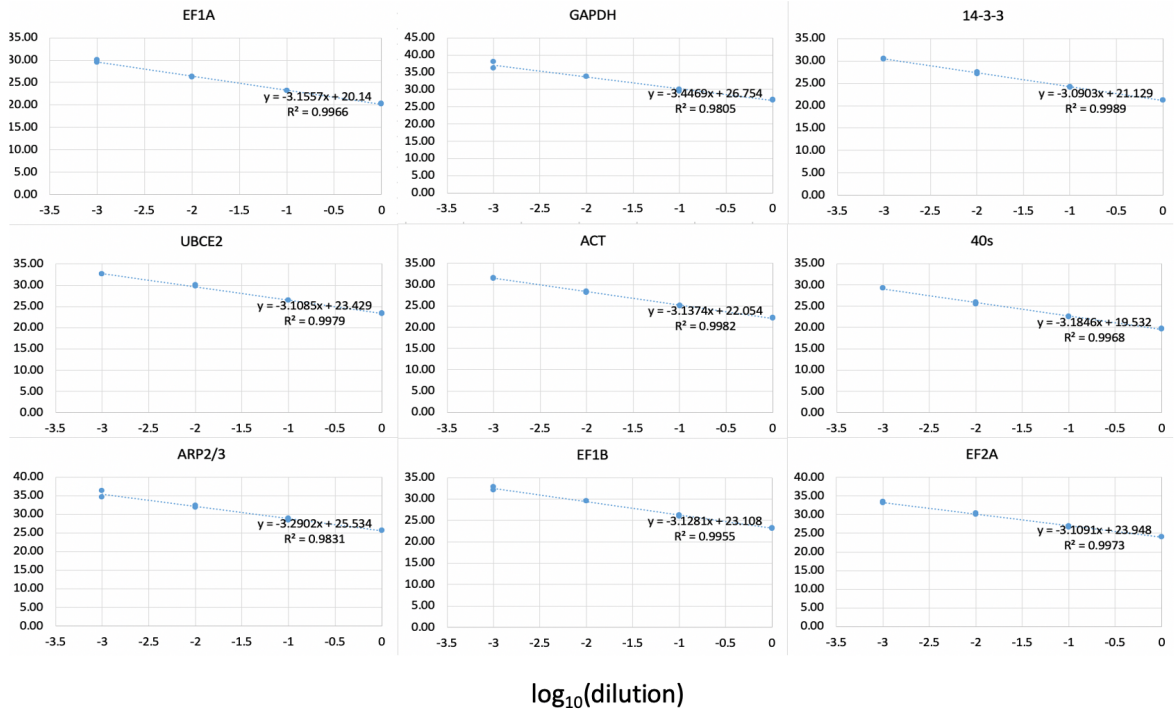

Supplement: S3 Fig — Amplification efficiency is determined from the slope of the log-linear portion of the calibration curve (y function coefficient). The initial template concentration (the independent variable; log10 of dilution 1x, 0.1x, 0.01x, 0.001x) is plotted on the x axis and corresponding Cq (the dependent variable) is plotted on the y axis. (PDF) [file pone.0233249.s003.pdf]

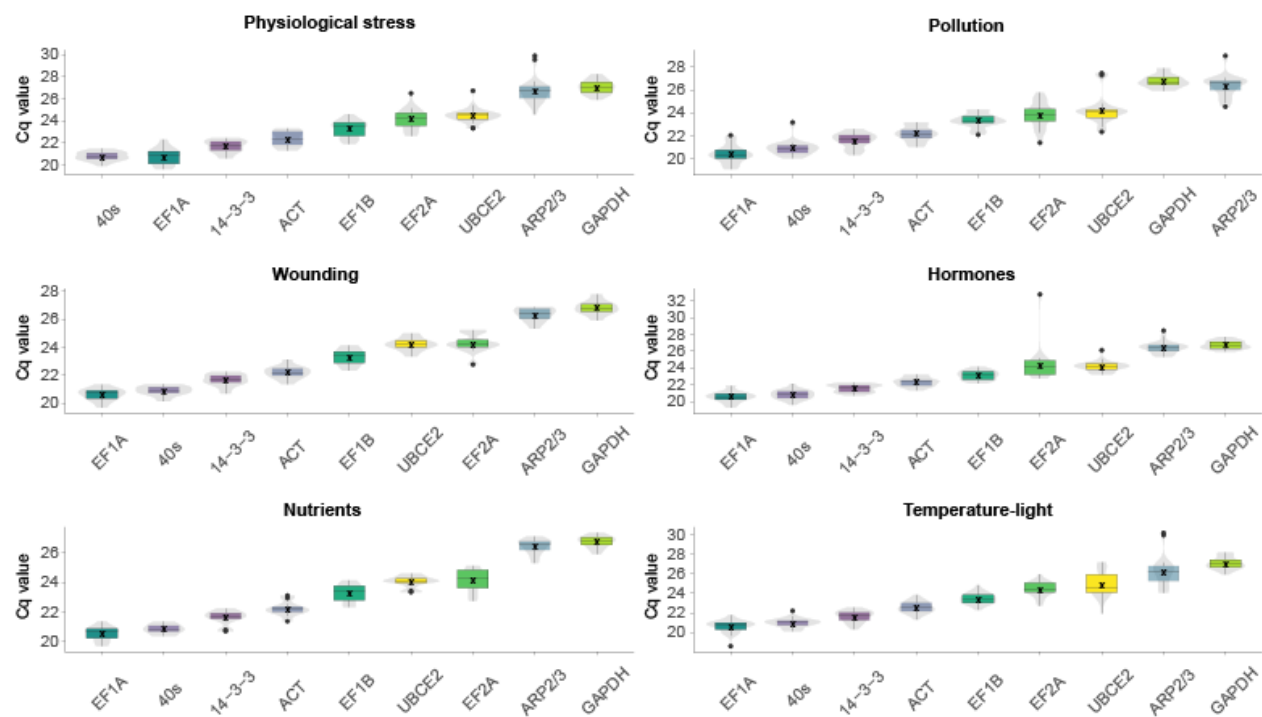

Supplement: S4 Fig — The boxplot marks the median (line) and 25th (lower) and 75th (upper) percentile; x marks the mean; the underlying violin plots show the data distribution for each housekeeping gene. Outliers are plotted as black dots. (PDF) [file pone.0233249.s004.pdf]

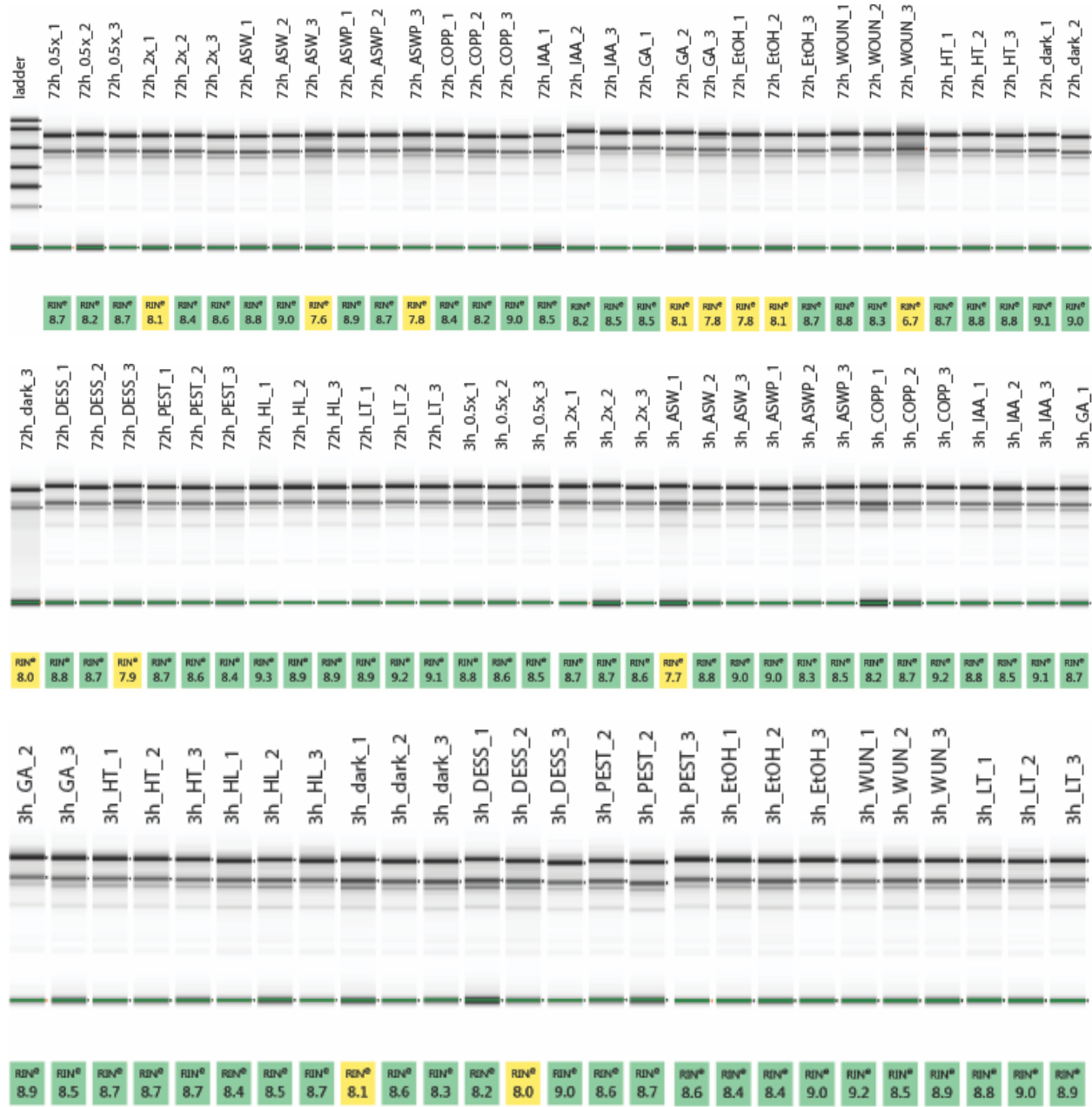

Supplement: S7 Fig — The High Sensitivity RNA ScreenTape assay was used for analyzing and assessing integrity of total RNA in all samples (following the manufacturer’s instructions) on a 2200 TapeStation Bioanalyzer (Agilent Technologies Inc.). RIN = RNA integrity number. (PDF) [file pone.0233249.s007.pdf]

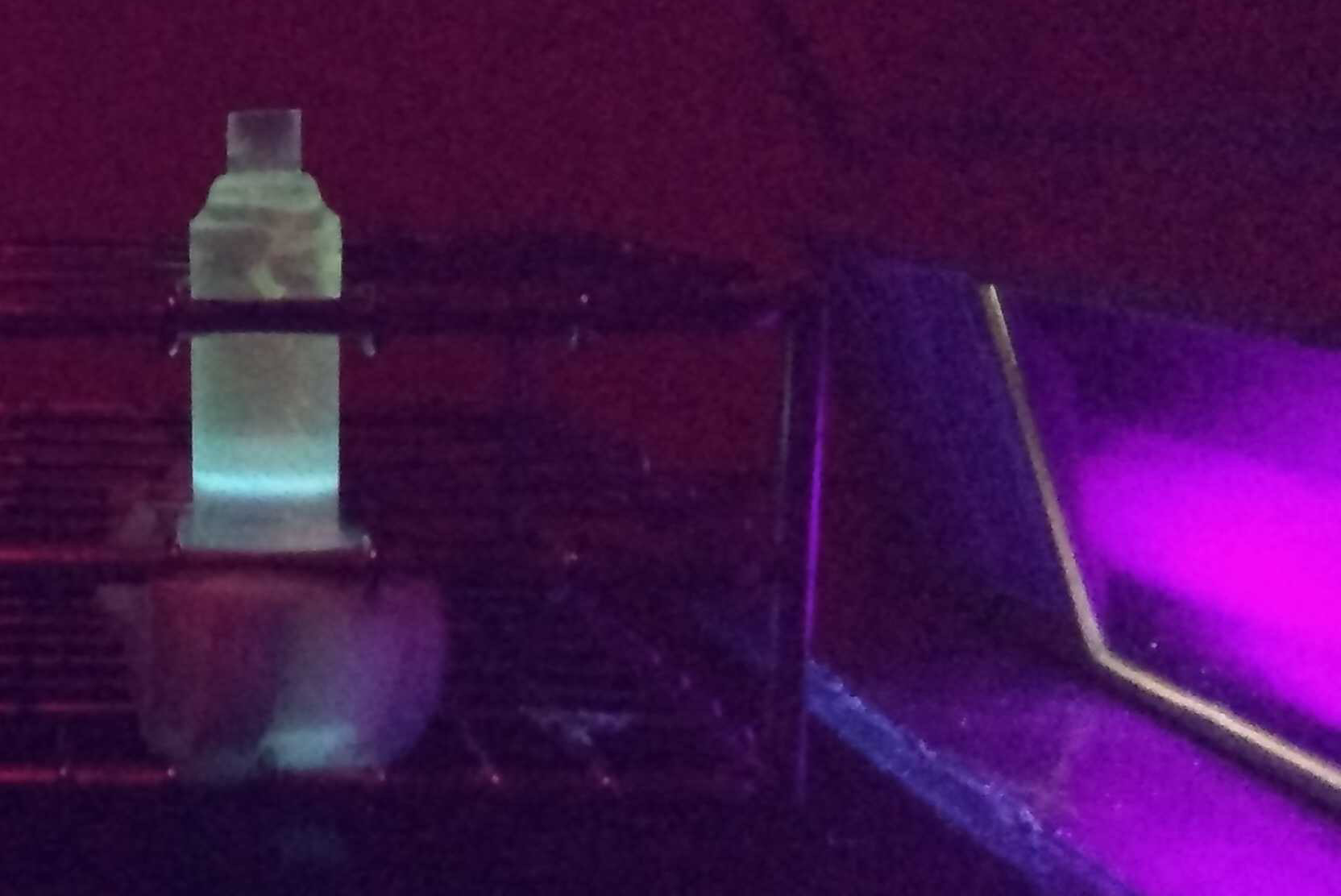

Supplement: S8 Fig — (JPG) [file pone.0233249.s008.jpg]
