# Supplementary material for: Identification and selection of optimal reference genes for qPCR-based gene expression analysis in Fucus distichus under various abiotic stresses
Source: PLoS One. 2021 Apr 28;16(4):e0233249. doi: 10.1371/journal.pone.0233249 (PMC8081170; doi:10.1371/journal.pone.0233249)
Supplement: S5 Fig — Stability values are are conditionally formatted (shade of color) according to their stability value, from the most stable (darkest shade) to the least stable (lightest shade). Purple corresponds to geNorm, green to NormFinder and yellow to BestKeeper analysis. Red text indicates genes over the threshold of acceptability, by algorithm. (PDF) [file pone.0233249.s005.pdf]

	physiological stress			pollution			wounding		
	**geNorm** *M-value*	**NormFinder** *SV*	**BestKeeper** *std dev [± CP]*	**geNorm** *M-value*	**NormFinder** *SV*	**BestKeeper** *std dev [± CP]*	**geNorm** *M-value*	**NormFinder** *SV*	**BestKeeper** *std dev [± CP]*
**EF1A**	0.41	0.37	0.60	0.75	0.34	0.58	0.37	0.24	0.36
**EF2A**	0.74	0.22	0.69	1.10	0.86	0.84	0.35	0.05	0.46
**EF1B**	0.59	0.15	0.62	0.36	0.15	0.46	0.29	0.19	0.46
**14-3-3**	0.63	0.03	0.47	0.65	0.16	0.52	0.64	0.23	0.34
**ARP2/3**	1.06	0.76	0.79	1.40	1	0.73	0.29	0.08	0.39
**UBCE2**	0.54	0.34	0.45	1.08	0.87	0.79	0.42	0.12	0.33
**40s**	0.65	0.37	0.33	0.81	0.48	0.45	0.48	0.22	0.28
**ACT**	0.44	0.3	0.59	0.55	0.36	0.52	0.99	0.14	0.65
**GADPH**	0.41	0.2	0.50	0.36	0.14	0.43	0.38	0.22	0.33

	hormones			nutrients			temperature - light		
	**geNorm** *M-value*	**NormFinder** *SV*	**BestKeeper** *std dev [± CP]*	**geNorm** *M-value*	**NormFinder** *SV*	**BestKeeper** *std dev [± CP]*	**geNorm** *M-value*	**NormFinder** *SV*	**BestKeeper** *std dev [± CP]*
**EF1A**	0.45	0.24	0.50	0.17	0.21	0.39	0.62	0.29	0.46
**EF2A**	1.98	0.98	1.19	0.61	0.29	0.66	0.39	0.1	0.54
**EF1B**	0.31	0.27	0.44	0.44	0.26	0.52	0.31	0.08	0.46
**14-3-3**	0.23	0.19	0.37	0.72	0.26	0.35	0.69	0.44	0.48
**ARP2/3**	0.65	0.07	0.52	0.17	0.05	0.42	1.48	1.23	1.06
**UBCE2**	0.47	0.11	0.51	0.31	0.14	0.27	1.25	1.24	1.01
**40s**	0.79	0.49	0.50	0.52	0.27	0.25	0.47	0.2	0.35
**ACT**	0.56	0.4	0.41	0.41	0.15	0.35	0.31	0.06	0.47
**GADPH**	0.23	0.07	0.44	0.32	0.18	0.32	0.54	0.29	0.49
