# Supplementary material for: Identification and selection of optimal reference genes for qPCR-based gene expression analysis in Fucus distichus under various abiotic stresses
Source: PLoS One. 2021 Apr 28;16(4):e0233249. doi: 10.1371/journal.pone.0233249 (PMC8081170; doi:10.1371/journal.pone.0233249)
Supplement: S6 Fig — (PDF) [file pone.0233249.s006.pdf]

gene	primer F 5’-3’	primer R 5’-3’
Hsp70	ACGCCAACGGTATCTTGAAC	CTCAATGCGTTGCCTCTGTA
Hsp90	CCTTTGCGAAGAACCTGAAG	TAAATACCGGCCTGCTTGTC

Hsp70

>Fucus transcriptome ‘gene’ CDS (TRINITY_DN38569_c4_g1_i1):

ATGGCAGCAGTGGATGGAGAGAGCGTAGGTATTGACCTTGGCACGACGTACTCCTGCGTCGGCGTGTGGCAAAACGA
CCGCGTCGAGATCATTGCCAATGACCAGGGTAACCGCACCACACCATCGTACGTGGCCTTTACCGAGACGGAGCGTC
TCATCGGCGACGCGGCTAAAAACCAGGTGGCGATGAACGCAACCAACACCGTGTTTGATGCCAAGCGGCTGATCGGC
CGTCGCTTCAGCGACCCCGCCGTGACGTCCGACAGGAAGCACTGGCCTTTCCACGTAGTAGAGGGACCCGGGGCGAA
GCCGACGATCGAGGTAATTTTCAAGGGCGAGAAGAAGCAGTTCGCTCCAGAGGAGATCTCATCGATGGTGCTCGTGA
AGATGAAGGAGATAGCTGAGGCCTACCTGGGCAAGGAGGTGAAGAACGCCGTGGTCACCGTGCCCGCGTACTTCAAC
GACTCCCAGCGACAAGCCACCAAGGATGCCGGTTCCATCGCCGGCCTTAATGTAATGCGAATCATCAACGAGCCCAC
AGCCGCCGCCATCGCGTATGGCCTCGACAAGAAGGGCGAAGAGAAGAACGTCCTCATCTTCGACCTCGGTGGCGGCA
CGTTCGACGTGTCTGTTCTCACGATCGAGGAAGGGATCTTCGAGGTTAAGGCCACCGCGGGAGACACTCACTTAGGA
GGGGAGGACTTCGACAACCGTATGGTGGACTACTTCCTGCAGGAGTTCAAGCGTAAATTCCGCAAGGACATGCGCTC
CAACAGCCGCGCGCTGCGACGTCTGCGCACGGCGTGCGAACGCGCGAAGCGTACGCTTTCGGCGTCGACCCAGGCGC
ACATCGAGATCGACTCGCTCTACGAGGGTATCGATTTCAACTCCACGATCACGCGCGCTAGGTTCGAGGACATGAAC
CAGGACTACTTCCAGAAGTGTCTGGCTCCGGTGGAGAAGGTTGTTAGGGACTCGAAGATGTCGAAGGGCCAGATCCA
CGAAGTGGTTCTGGTGGGCGGGTCCACTCGCATTCCCAAGATCCAGCAGATGCTGGTGGACTACTTCAACGGTAAGG
AACCGTGCAAGTCCATCAACCCCGACGAGGCCGTGGCCTACGGCGCCACCGTCCAGGCCGCCATCCTCTCTGGCCAG
GACAAGTCCGACAAGCTCAACTCCCTCCTGCTGCTGGACGTGACCCCTCTGGGCTTCGGTCTGGAGACGGCCGGCGG
CGTGATGACCACGCTCATCAAGCGGAATACTACCGTGCCGGCGAAGAAATCTCAGGTTTTCTCGACCTACGCCGACA
ACCAGCCCGGGGTGCTTATCCAGGTGTACGAGGGAGAGCGCACCATGACCCGTGACAACAACCTTTTGGGCAAGTTC
AACCTGGACGGCATCCCGCCTATGCCTCGCGGGCAGCCGCAGATCGAGGTGACGTTCGACATCGACGCCAACGGTAT
CTTGAACGTGCACGCGGCGGAGAAATCTACGGGCAAGGAGAACAAGATCACAATCACGAACGACAAGGGCCGTCTGA
GCGCGGATGAGATCGAGCGCATGGTGGAGGAGGCGGAGCGGTACAAGGCGGAGGACGACGTACAGAGGCAACGCATT
GAGGGCAAGAATGCTCTGGAGAACTACGCGTACTCGATGCGCAACACGATCAACGAGGAGAAGGTTGCCTCGCAGCT
GGACGCAGCGGACAAGTCGGCGATCGAGAGCAAGATCTCGGAGACGATCGCGTGGCTGGACGCGAACCAGACCGCGG
AGAAAGAAGAGTACGAAGAGAAGCAGAAGGAGCTCGAGGGCGTATGCACCCCCATCATCCAGAAGATGGCAGCCGCG
GGTGGCGGTGGTATGCCCGGTGGCATGGGTGGCATGCCGGACATGGGCGGTGCTGGTTTCCCCGGCGCGGGGGCACC
TACGCCAGAGGCTGATGCCGACGCTGGCCCGAAGATCGAGGAGATTGACTAG

Hsp90

>Fucus transcriptome ‘gene’ CDS (TRINITY_DN38044_c1_g7_i1):

ATGTCTGAACCTGCGGCAAACCCTACGCCTGAAGCGCCTGAGGCGTCTGAGGCGGAGACCTTCGCTTTCTCCGCTGA
TATCAACCAGCTGCTCTCCCTTATCATCAACACCTTCTACTCCAACAAGGAGATTTTCCTTCGAGAGATCATCTCCA
ACAGCAGTGATGCACTCGACAAGATCCGATATCAGTCGTTGACTGACAAGGCTGTGCTTGACTCAGAGCCCAACATG
GAGATCCGCATCATCCCTGACAAGGCGAACAACACCTTGACCATCGAGGACACCGGAATTGGAATGACGAAGGCTGA
CCTCGTTAACAACCTGGGAACGATCGCTAAGTCCGGCACGAAGGCGTTCATGGAGGCACTTAGCGCCGGGGCGGACA
TCAGCATGATCGGGCAATTCGGCGTGGGTTTCTACTCCGCTTACCTGGTGGCTGACAAGGTGACTGTTACCTCCAAG
AGCAACGACGACGAGCAGCACACCTGGGAGTCCTCCGCCGGCGGTTCATTCACCGTCACCCAGGACGGCCCCGACGC
GAAGCCCCTTGGGCGAGGAACTCGCATCGAGCTCATCCTGAAGGAGGACATGGCAGAATTTCTCGAGGAGAGGAAGG
TCAAGGACCTAGTGAAAAAGCACTCGGAGTTCATTGGTTTCCCGATCAAGTTGTACACGGAGAAGACTACGGAGAAG
GAGGTGACGGACGATGAGGACGAGGAGGAGGCCGAAGAGGGTGACGAGGACAAGGAGCCCAAGGTGGAAGATGTTGA
CGAGGAAGAGACGAAGGACAAGAAGACAAAGAAGATTAAAGAGGTGTCTCACGAGTGGGACCACCTGAACCAGCAGA
AGCCGATCTGGATGCGAAAGTCTGACGAAGTCACTCACGACGACTACGCCGCCTTCTACAAGTCGCTCAGCAACGAC
TGGGAGGACCACGCGGCTGTAAAGCACTTCTCCGTGGAGGGACAGCTCGAGTTCCGATCCGTACTCTTCGTGCCCAA
GCGCGCTCCGTTCGACATGTTCGAGGGAGGAACAAAGAAGAAGCACAACAACATCAAACTGTACGTACGTCGCGTGT
TTATCATGGACAACTGTGAGGACTTGATGCCCGAGTACCTGCAGTTCGTGAAGGGCGTGGTGGACTCCGAGGACCTG
CCGTTGAACATCTCGCGTGAGAGTCTTCAGCAGAACAAGATCCTCCGCGTTATCCGCAAGAACTTGGTGAAGAAATC
CGTTGAGCTTTTCAACGAGCTTGCCGAGGACGCGGAAAAGTACAAAAAGTTCTACGAAGCCTTTGCGAAGAACCTGA
AGCTCGGCATCCACGAAGACTCAACCAACCGTGCCAAGCTGGCGAAGCTCCTTCGGTACCACTCGACGAAGAGCGGC
GAGGAGATGACGTCACTCGATGACTATGTGGCGAGAATGGACGACAAGCAGGCCGGTATTTACTATGTGACGGGCGA

GTCTAAGATGGCTGTCGAGAACTCTCCTTTCCTGGAGAAGCTAAAGAAGAAGGGGGTCGAGGTGCTTTTTATGGTCG
ATCCCATTGACGAGTACGCTGTCCAGCAGCTCAAGGAGTTCGAGGGAAAGAAGCTTATCTGCGCTACCAAAGAGGGA
ATGAAAATCGATGAGTCTGACGACGAGGCGAAGAAGTTTGAGGAGGCAAAGGCGGCTTCGGAAGGCCTGTGCAAGCT
GATGAAGGAGGTGCTGGCGGACAAGGTAGACAAGGTGGTGGTGTCGAACCGTCTGGCGGATTCTCCGTGCGTGTTGG
TGACTGGAGAGTACGGATGGTCGGCAAACATGGAGCGCATCATGAAGGCGCAGGCGCTTCGTGATTCTTCGACGTCG
TCCTACATGACTTCGAAGAAGACCATGGAGGTCAACCCCACCAACTCCATCGTTGTTGCGCTCCGAGAGAAGGCATC
TGCGGACCAAAGCGACAAGACGGTGAAGGACCTGATCTGGCTGCTGTACGACACCTCCCTGCTCACTTCAGGGTTCA
GCCTCGACGAGCCTACCACCTTTGCGGGTCGCATTCACAGGCTTATCAAGTTGGGTCTGTCCATCGATGAGGATGAT
GCAGCAGGAGACGACGGAGAGGACGACATTCCCGACCTCGATGACGCCGAAGGAGACGAAGAGTCCACCATGGAGCA
AGTCGACTGA
