# Supplementary material for: Identification and selection of optimal reference genes for qPCR-based gene expression analysis in Fucus distichus under various abiotic stresses
Source: PLoS One. 2021 Apr 28;16(4):e0233249. doi: 10.1371/journal.pone.0233249 (PMC8081170; doi:10.1371/journal.pone.0233249)
Supplement: S1 Table — Column 1 –treatment, column 2 –parameters of the treatment, column 3 –length of treatment, column 4 –associated group of treatments. (PDF) [file pone.0233249.s009.pdf]

	Treatment	Parameters	Treatment length	Condition group
**1**	Artificial seawater (ASW)	16C,<br>12:12 light:dark,<br>~60 µmol photons m^{-1} s^{-1}	3h and 72h	Nutrients
**2**	Artificial seawater (ASW) + Provasoli Enrichment (P)	16C,<br>12:12 light:dark,<br>~60 µmol photons m^{-1} s^{-1}	3h and 72h	Control; Nutrients, Physiological stress, Pollution, Hormones, Wounding, Temperature-light
**3**	Low salinity (0.5x ASW)	16C,<br>12:12 light:dark,<br>~60 µmol photons m^{-1} s^{-1}	3h and 72h	Physiological stress
**4**	High salinity (2x ASW)	16C,<br>12:12 light:dark,<br>~60 µmol photons m^{-1} s^{-1}	3h and 72h	Physiological stress
**5**	Desiccation	16C,<br>12:12 light:dark,<br>~60 µmol photons m^{-1} s^{-1}	3h and 72h	Physiological stress
**6**	Wounding	16C,<br>12:12 light:dark,<br>~60 µmol photons m^{-1} s^{-1}	3h and 72h	Wounding
**7**	Indole-3-acetic acid (auxin, IAA)	16C,<br>12:12 light:dark,<br>~60 µmol photons m^{-1} s^{-1}<br>50µM	3h and 72h	Hormones
**8**	Gibberelic acid (GA)	16C,<br>12:12 light:dark,<br>~60 µmol photons m^{-1} s^{-1}<br>50µM	3h and 72h	Hormones
**9**	Ethanol (control for IAA and GA)	16C,<br>12:12 light:dark,<br>~60 µmol photons m^{-1} s^{-1}	3h and 72h	Hormones
**10**	$CuSO_4$	16C,<br>12:12 light:dark,<br>~60 µmol photons m^{-1} s^{-1}<br>10µM	3h and 72h	Pollution
**11**	Pesticide (Imidacloprid)	16C,<br>12:12 light:dark,<br>~60 µmol photons m^{-1} s^{-1}<br>0.1ug/L	3h and 72h	Pollution
**12**	Dark	16C,<br>dark	3h and 72h	Temperature-light
**13**	High light	16C,<br>12:12 light:dark,<br>~120 µmol photons m^{-1} s^{-1}	3h and 72h	Temperature-light
**14**	Low temperature	8C,<br>12:12 light:dark,<br>~60 µmol photons m^{-1} s^{-1}	3h and 72h	Temperature-light
**15**	High temperature	22C,<br>12:12 light:dark,<br>~60 µmol photons m^{-1} s^{-1}	3h and 72h	Temperature-light
