# Supplementary material for: Identification and selection of optimal reference genes for qPCR-based gene expression analysis in Fucus distichus under various abiotic stresses
Source: PLoS One. 2021 Apr 28;16(4):e0233249. doi: 10.1371/journal.pone.0233249 (PMC8081170; doi:10.1371/journal.pone.0233249)
Supplement: S2 Table — (PDF) [file pone.0233249.s010.pdf]

ITEM TO CHECK	IMPORTANCE	CHECKLIST
**EXPERIMENTAL DESIGN**		
Definition of experimental and control groups	**E**	x
Number within each group	**E**	x
Assay carried out by core lab or investigator's lab?	D	x
Acknowledgement of authors' contributions	D	x
**SAMPLE**		
Description	**E**	x
Volume/mass of sample processed	D	x
Microdissection or macrodissection	**E**	x
Processing procedure	**E**	x
If frozen - how and how quickly?	**E**	x
If fixed - with what, how quickly?	**E**	NA
Sample storage conditions and duration (especially for FFPE samples)	**E**	x
**NUCLEIC ACID EXTRACTION**		
Procedure and/or instrumentation	**E**	x
Name of kit and details of any modifications	**E**	x
Source of additional reagents used	D	x
Details of DNase or RNAse treatment	**E**	x
Contamination assessment (DNA or RNA)	**E**	x
Nucleic acid quantification	**E**	x
Instrument and method	**E**	x
Purity (A260/A280)	D	x
Yield	D	
RNA integrity method/instrument	**E**	x
RIN/RQI or Cq of 3' and 5' transcripts	**E**	x
Electrophoresis traces	D	x
Inhibition testing (Cq dilutions, spike or other)	**E**	x
**REVERSE TRANSCRIPTION**		
Complete reaction conditions	**E**	x
Amount of RNA and reaction volume	**E**	x
Priming oligonucleotide (if using GSP) and concentration	**E**	x
Reverse transcriptase and concentration	**E**	x
Temperature and time	**E**	x
Manufacturer of reagents and catalogue numbers	D	x
Cqs with and without RT	D*	
Storage conditions of cDNA	D	x
**qPCR TARGET INFORMATION**		
If multiplex, efficiency and LOD of each assay.	**E**	NA
Sequence accession number	**E**	NA (no number)
Location of amplicon	D	
Amplicon length	**E**	x
In silico specificity screen (BLAST, etc)	**E**	x
Pseudogenes, retropseudogenes or other homologs?	D	
Sequence alignment	D	
Secondary structure analysis of amplicon	D	

Location of each primer by exon or intron (if applicable)	**E**	NA
What splice variants are targeted?	**E**	NA
**qPCR OLIGONUCLEOTIDES**		
Primer sequences	**E**	x
RTPrimerDB Identification Number	D	
Probe sequences	D**	
Location and identity of any modifications	**E**	NA
Manufacturer of oligonucleotides	D	IDT
Purification method	D	
**qPCR PROTOCOL**		
Complete reaction conditions	**E**	x
Reaction volume and amount of cDNA/DNA	**E**	x
Primer, (probe), Mg++ and dNTP concentrations	**E**	x
Polymerase identity and concentration	**E**	x
Buffer/kit identity and manufacturer	**E**	x
Exact chemical constitution of the buffer	D	
Additives (SYBR Green I, DMSO, etc.)	**E**	x
Manufacturer of plates/tubes and catalog number	D	
Complete thermocycling parameters	**E**	x
Reaction setup (manual/robotic)	D	manual
Manufacturer of qPCR instrument	**E**	x
**qPCR VALIDATION**		
Evidence of optimisation (from gradients)	D	
Specificity (gel, sequence, melt, or digest)	**E**	x
For SYBR Green I, Cq of the NTC	**E**	x
Standard curves with slope and y-intercept	**E**	x
PCR efficiency calculated from slope	**E**	x
Confidence interval for PCR efficiency or standard error	D	
r2 of standard curve	**E**	x
Linear dynamic range	**E**	x
Cq variation at lower limit	**E**	x
Confidence intervals throughout range	D	
Evidence for limit of detection	**E**	x
If multiplex, efficiency and LOD of each assay.	**E**	NA
**DATA ANALYSIS**		
qPCR analysis program (source, version)	**E**	x
Cq method determination	**E**	x
Outlier identification and disposition	**E**	x
Results of NTCs	**E**	x
Justification of number and choice of reference genes	**E**	x
Description of normalisation method	**E**	x
Number and concordance of biological replicates	D	x
Number and stage (RT or qPCR) of technical replicates	**E**	x
Repeatability (intra-assay variation)	E	x
Reproducibility (inter-assay variation, %CV)	D	
Power analysis	D	

Statistical methods for result significance	**E**	x
Software (source, version)	E	x
Cq or raw data submission using RDML	**D**	
