# Supplementary material for: Identification and selection of optimal reference genes for qPCR-based gene expression analysis in Fucus distichus under various abiotic stresses
Source: PLoS One. 2021 Apr 28;16(4):e0233249. doi: 10.1371/journal.pone.0233249 (PMC8081170; doi:10.1371/journal.pone.0233249)
Supplement: Attachment — Submitted filename: Response to Reviewers.docx [file pone.0233249.s011.docx]

*Linardić and Braybrook – Identification and selection of optimal reference genes for qPCR-based gene expression analysis in Fucus distichus under various abiotic stresses*

We thank the editor and reviewer for their careful reading of our manuscript. We are pleased to provide a corrected manuscript, with the details of changes outlined below.

***RESPONSE TO REVIEWERS***

***Editor comments:***

1. *Parameters applied for RankAggreg (CE Kendall or CE Spearman? Weight settings? What stopping criteria?) to ensure replicability.*

**RESPONSE**:

Added parameters used in Materials and Methods as follows at line 497:

‘In addition, a rank aggregation method based on a Monte Carlo cross-entropy algorithm (R-package; RankAggreg 0.6.5; https://CRAN.R-project.org/package=RankAggreg; with default settings and following set parameters: method= "CE", distance= ”Spearman”, convIn= 7, seed=100) was used to combine the gene ranks of the three above algorithms and create a consensus housekeeping gene ranking.’

2. *A MIQE checklist (*[*https://rdml.org/miqe.html*](https://rdml.org/miqe.html)*) should be added as supplement information and experimental details supplied accordingly.*

**RESPONSE**:

Added to the Supplement as Table S2.

***Reviewer comments:*** *in the text, such as:
Line 158, there are two spaces among genes pooled
Line 178, Fig. 2ABC should be Fig. 2A-C
Line 389, the two initial sentences in conclusion are not conclusions of this work, please delete
Line 405, 450 space mM, 10 space mM and all over M&M
Line 413, 0.2 space μL
Line 432, change V to v for volumen
Line 434, change EtOH to ethanol
Line 445, change NaOAc to ammonium acetate
Line 455 change 10 ng/uL to 10 ng/μL
Revise reference format , there are several tipos
Ref 8 in the number of the article
Ref 13 Franco P-O should be Franco PO, and Le Bail A
Ref 15 Extremophiles. Delete colon
Ref 36 is a journal bioRXIV?
Ref52 delete capital letters in the title as well as in ref 53 and 54
Ref 62. Delete MGG before Mol, Gen Genet*

**RESPONSE**:

Line 389 – removed first sentence, but kept second sentence due to better introduction to our conclusions.

Ref 36 – BioRxiv is a preprint server:

(from PLOS Submission guidelines: *“Any and all available works can be cited in the reference list. Acceptable sources include:*

- *Published or accepted manuscripts*
- *Manuscripts on preprint servers, providing the manuscript has a citable DOI or arXiv URL.”*)

Corrected all typos and errors.
